# *Fusobacterium necrophorum* Brain Abscess Following Invasive Sinusitis in an Immunocompetent Adult: A Case Report

**DOI:** 10.5811/cpcem.33523

**Published:** 2025-02-15

**Authors:** Andres V. Somoza, Christina T. Hanos, Jesse W. St Clair, Courtney L. James

**Affiliations:** * Mayo Clinic Alix School of Medicine, Mayo Clinic College of Medicine and Science, Jacksonville, Florida; † Department of Family Medicine, Mayo Clinic School of Graduate Medical Education, Mayo Clinic College of Medicine and Science, Jacksonville, Florida; ‡ Department of Emergency Medicine, Mayo Clinic, Jacksonville, Florida

## Abstract

**Introduction:**

A brain abscess is a localized collection of purulent infection within the brain parenchyma. It most often occurs due to contiguous spread from sinus, otogenic, and odontogenic infections; however, it can also develop from direct intracranial contact via trauma or surgery. *Fusobacterium necrophorum*, an obligate anaerobic, gram-negative bacillus, is part of the normal flora of the oral cavity. Given its inherent location, F *necrophorum* has been shown to contribute to complications stemming from infection of the tonsils, pharynx, and teeth. Invasive infections of F necrophorum are seldomly seen in immunocompetent patients.

**Case Report:**

We report a case of a previously healthy 20-year-old man who presented to our emergency department with headache, facial pain, and neck stiffness. He was ultimately found to have an *F necrophorum* intracranial abscess and underwent right frontal craniotomy with evacuation of epidural abscess and partial sinus obliteration. He was placed on broad-spectrum antibiotics, including vancomycin, cefepime, and metronidazole for six weeks. His treatment course was complicated by recurrence of intraparenchymal abscess requiring repeat craniotomy with abscess evacuation and advancement of antibiotic regimen to meropenem. To our knowledge, there are no reported cases in the literature of monomicrobial *F necrophorum* brain abscesses arising secondary to invasive sinusitis in immunocompetent adults.

**Conclusion:**

This report highlights the clinical presentation, diagnostic strategies, management challenges, clinical outcomes, and complications of invasive sinusitis leading to brain abscess formation in an otherwise healthy adult male.

## INTRODUCTION

A brain abscess is a localized collection of purulent infection within the brain parenchyma. It most often occurs from contiguous spread from sinus, otogenic, and odontogenic infections; however, it can also develop due to direct intracranial contact via trauma or surgery.^1^–^3^ Intracranial abscesses typically contain aerobic and anaerobic bacteria, with anaerobes as the more common isolate—specifically, the gram-negative Fusobacterium species.^4^ In instances where Fusobacterium brain abscesses or sinusitis are present, it is typical to observe the growth of *Fusobacterium nucleatum* species in cultures.^5^–^8^ In contrast, the identification of *Fusobacterium necrophorum* in isolates is relatively rare.^6^,^7^ Apart from sinusitis and brain abscesses, *F necrophorum* is a well-documented cause of a variety of other infections, including Lemierres syndrome, pharyngitis, meningitis, and septicemia. This bacterium can be found as part of the normal microbial flora of the mouth and throat in healthy individuals and can therefore contribute to invasive complications stemming from infection of the tonsils, pharynx, and teeth. However, invasive infections of *F necrophorum* are seldom seen in immunocompetent patients.^7^,^9^

We report a case of a previously healthy man who presented to our emergency department (ED) with headache and was found to have an *F necrophorum* intracranial abscess. To our knowledge, there are no reported cases in the literature of monomicrobial *F necrophorum* brain abscesses arising secondary to invasive sinusitis in immunocompetent adults. Previously associated with high incidence of mortality, the outcomes for those with brain abscesses have improved with the advent of computed tomography (CT) and magnetic resonance imaging (MRI) (0.4–0.9 cases per 100,000).^10^,^11^ However, despite these advancements, untimely identification and treatment continue to result in poor outcomes.^12^ Thus, our work aims to highlight the clinical presentation, diagnostic strategies, management challenges, clinical outcomes, and complications of this rare infection.

## CASE REPORT

A 20-year-old man with no significant past medical history presented to the emergency department (ED) for evaluation of fever, headache, neck pain and stiffness, and facial pain. In the preceding month, he was initially diagnosed with influenza, which improved with supportive treatment until two weeks into his influenza diagnosis, when he began experiencing increased facial pain and intermittent fevers. For these new symptoms, he was diagnosed with bacterial sinusitis and was given a seven-day course of amoxicillin/trimethoprim which he took to completion. Symptoms initially resolved during the course of antibiotics, but by the end his symptoms returned. He represented to his primary care physician and was started on doxycycline. After three days of doxycycline, he presented to the ED with worsening headaches exacerbated by movement, neck pain and stiffness with flexion, continued facial pain, and intermittent fevers. He reported minimal relief from over-the-counter analgesics and antipyretics. He was fully vaccinated and had no recent wilderness exposure, insect bites, or travel.

In the ED, vital signs included a temperature of 37.8 °Celsius, heart rate of 109 beats per minute, respirations at 16 breaths per minute, blood pressure of 128/66 millimeters of mercury, and oxygen saturation of 100% on room air. On examination, he appeared uncomfortable and was wearing sunglasses due to photophobia. Inspection of the head and face revealed normal appearance without significant lesions. Palpation of the face and sinus cavities were positive for tenderness bilaterally. There was no salivary gland swelling or tenderness. Otoscopic exam revealed normal external auditory canals and tympanic membranes. Nasal inspection displayed normal nasal mucosa without evidence of septum abnormality. Oral inspection revealed oropharynx with normal mucosa and oral cavity negative for asymmetry, lesions, masses, erythema, exudates. Although his neck was no rigid, he had some stiffness and pain with flexion. Kernig and Brudzinski signs were negative, and neurologic examination revealed no focal deficits. He was alert and oriented to person, place, and time.

**CPC-EM Capsule**

What do we already know about this clinical entity?

Fusobacterium necrophorum *is often linked to sinusitis or Lemierre’s syndrome in immunocompromised patients and rarely causes brain abscesses*.

What makes this presentation of disease reportable?

*This case is the first reported monomicrobial F. necrophorum brain abscess secondary to invasive sinusitis in an immunocompetent adult*.

What is the major learning point?

*Early recognition and aggressive multidisciplinary management of atypical pathogens like F. necrophorum can prevent severe complications and improve outcomes*.

How might this improve emergency medicine practice?

*Enhances awareness of rare brain abscess etiologies and emphasizes timely imaging and treatment for atypical, persistent sinusitis symptoms*.

**Image 1:**
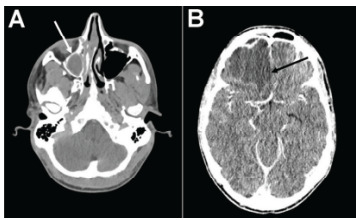
Head computed tomography without (A) and with (B) intravenous contrast. Imaging demonstrated a complicated right frontal sinusitis (white arrow) with intracranial extension and formation of an abscess with extensive associated vasogenic edema and mass effect with two-millimeter leftward midline shift (black arrow).

While awaiting further tests, the patient received intravenous (IV) fluids, analgesics, and antiemetics and received empiric treatment for meningitis with IV vancomycin, ceftriaxone, and dexamethasone. Initial laboratory studies revealed leukocytosis with 17.3 x 109 white blood cells per liter (L) (reference range: 3.4–9.6 x 109 cells/L) with neutrophilia of 94.8% (50.0–75.0%), C-reactive protein of 20.6 milligrams (mg)/L (<5.0 mg/L), and lactate of 1.7 millimoles (mmol)/L (0.5–2.2 mmol/L). Head CT with and without contrast demonstrated complicated right frontal sinusitis with intracranial extension and formation of an epidural abscess with extensive surrounding vasogenic edema and leftward midline shift (Image 1). A neurosurgeon was emergently consulted, and antibiotic coverage was broadened to IV vancomycin, cefepime, and metronidazole.

The patient underwent emergent right frontal craniotomy for evacuation of epidural abscess and obliteration of the connection between the frontal sinus and intracranial compartment. Bacterial cultures and Gram stain of a sample from the intracranial abscess were obtained, and the patient was admitted to the neurointensive care unit. On hospital day three, an otorhinolaryngologist performed a functional endoscopic sinus surgery with right maxillary antrostomy, right total ethmoidectomy, right sphenoidotomy, and right frontal sinusotomy with additional washout. During the procedure, the patient was noted to have severe inflammation of the mucosa of the ethmoid bulla and anterior ethmoidal cells, which appeared to be consistent with an ascending infection. Intraoperative cultures of purulent drainage from the maxillary and frontal sinuses were collected. At this time, the culture sample from the intracranial abscess showed growth of *F necrophorum*, so a peripherally inserted central catheter was placed in anticipation of long-term antibiotics.

On day of discharge, samples from the sinus showed no growth. The patient had marked improvement, and his leukocyte count was down to 8.7 x 10^9^ cells/L. He was sent home on a corticosteroid taper, six weeks of oral metronidazole 500 mg three times daily, and six weeks of IV ceftriaxone 2 grams (g) twice daily with planned infectious disease outpatient clinic follow-up.

**Image 2:**
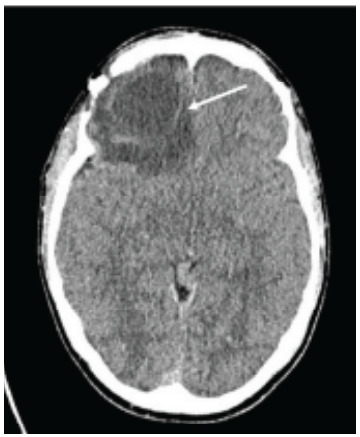
On the patient’s second emergency department visit for recurrence of symptoms, head computed tomography demonstrated interval development of a hypodense focus with hyperattenuating rim, concerning for a recurrent organizing intraparenchymal abscess with two millimeters leftward midline shift (arrow).

**Image 3:**
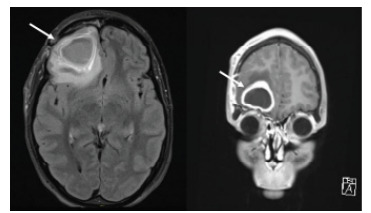
On the patient’s second emergency department visit for recurrence of symptoms, magnetic resonance imaging of the brain with contrast demonstrated recurrent large right inferior frontal lobe cerebral abscess with significant surrounding vasogenic edema.

Approximately one month after discharge, the patient returned to the ED due to severe headache, retro-orbital pain, and intractable nausea and vomiting. Computed tomography without contrast and MRI with contrast of the head demonstrated intraparenchymal abscess and moderate lobulated opacification of the right maxillary sinus (Images 2 and 3). He was subsequently admitted to the neurointensive care unit and underwent repeat right frontal craniotomy for abscess evacuation. Home antibiotics of metronidazole and ceftriaxone were continued in their IV formulations, with the addition of vancomycin for broader coverage. Levetiracetam and dexamethasone were also added, and the patient was discharged five days later with close neurosurgical, otorhinolaryngologic, and infectious disease follow-up. At discharge, he was afebrile, hemodynamically stable, and free of retro-orbital pain, nausea, vomiting, and headache.

He returned to the ED ten days later with complaints of increased headache, fever, photophobia, intermittent bilateral hand numbness, and nausea. Given his history of brain abscess with recurrence, the patient was admitted to the neurosurgery service. Head CT with and without contrast and brain MRI with and without contrast demonstrated stable postsurgical changes without any abscess development. The infectious disease specialists advanced the antibiotic regimen to two g of IV meropenem every eight hours. The patient demonstrated clinical improvement and was discharged home three days later with close follow-up with specialty services and made a complete recovery in the outpatient setting. Brain MRI obtained two months after initial presentation revealed continued evolution of postsurgical changes within the right frontal region with no findings suggesting recurrent or progressive abscess.

## DISCUSSION

Treatment of brain abscesses involves a multidisciplinary approach with various surgical and medical interventions depending on the infection source and microorganisms identified. With proper treatment, the overall mortality rate for brain abscesses has decreased to around 5% to 10%.^10^ In this case, the positive culture for *F necrophorum* guided the selection of antibiotics. The patient’s immunocompetent status, despite the severity of the infection, may have contributed to his resilience during treatment.

The conventional risk factors associated with the development of brain abscesses encompass previous head trauma, recent neurosurgical procedures, or persistent infections (such as otitis media, dental caries, and sinusitis) that may facilitate contiguous dissemination. Specific populations at heightened risk include immunocompromised patients (eg, HIV/AIDS), those suffering from chronic pulmonary diseases, and patients with diabetes mellitus or certain neoplastic disorders. Additionally, individuals traveling internationally may encounter infections like cysticercosis, which can lead to abscess formation.^1^,^4^

Regarding prognosis, early diagnosis and comprehensive treatment are associated with favorable outcomes, particularly in patients who present with preserved consciousness on admission.^5^ However, the risk of recurrence of brain abscesses is high, with reports indicating a rate of approximately 10%, as seen in our case. Moreover, the progression from sinusitis to brain abscess is a rare and severe complication, occurring in approximately 0.4% of patients with sinusitis, particularly in those with untreated or inadequately managed bacterial sinusitis.^10^ This statistic highlights the need for long-term follow-up and diligent monitoring, even for those without traditional risk factors.^11^

Emergency physicians should maintain high suspicion for brain abscesses in patients presenting with undifferentiated headache and sinus pressure, especially when accompanied by neurological signs such as neck stiffness, fever, or changes in mental cognition. Neuroimaging should be obtained in these cases to rule out brain abscess, especially when symptoms persist despite initial treatment for sinusitis or if there is severe systemic illness. Computed tomography is typically the first-line imaging modality due to ease and rapidity of results, but MRI is preferred for its increased sensitivity in diagnosing, monitoring, and characterizing the development of brain abscesses.^1^ However, the time and cost associated with MRI may limit optimal care for some individuals. Another challenge in managing brain abscesses is identifying the causative microorganism and determining appropriate treatment. Common microorganisms include *Staphylococcus aureus*, Streptococcus species, and anaerobic bacteria like Fusobacterium.^1^,^2^ Initial empirical antibiotic therapy should cover these, often starting with vancomycin for gram-positive coverage, combined with ceftriaxone or cefepime for broad-spectrum gram-negative coverage, and metronidazole for anaerobes.^2^ In cases where *F necrophorum* is identified, as in this patient, treatment may be escalated to include meropenem or clindamycin based on susceptibility patterns.

In our patient, *F necrophorum* was identified through bacterial cultures and Gram stain of a cranial abscess sample. The isolation of this common oral flora from the cranial abscess and severe inflammation observed in the ethmoid sinus during surgery confirmed that the infection ascended from the sinuses to the brain. Bacterial cultures from intracranial surgery revealed the organism was susceptible to clindamycin, metronidazole, and penicillin, a typical susceptibility pattern for this bacterium species. Despite appropriate initial antibiotic therapy, the patient had symptom recurrence. This is believed to have been due to worsening inflammation from corticosteroid discontinuation rather than worsening infection from inadequate antibiotics because there was no abscess recurrence and repeat craniotomy cultures remained negative. Nonetheless, the patient was escalated to higher-intensity antibiotics and additional corticosteroids per neurosurgical recommendation. This case highlights a gap in our understanding of the pathogenesis of *F necrophorum* and proposed duration of the resultant cerebral inflammation, which is a potential area for further research.

Also of interest is the lack of growth on the initial sinus cultures, which may have occurred due to method of culturing or partial response of the infection to previously administered antibiotics.^8^ In cases such as these, metagenomic next generation sequencing has been shown to rapidly reveal underlying microorganisms with reduced impact from prior antibiotic exposure.^13^

## CONCLUSION

This case highlights the complexities involved in diagnosing and managing sinusitis and its potential complications in healthy individuals. The insidious onset of symptoms, such as headache and fever without meningeal signs, can mimic other intracranial pathologies, leading to delays in diagnosis and management. Given risk of neurologic deterioration secondary to mass effect and shift changes with elevated intracranial pressure, CT should be obtained prior to considering lumbar puncture in patients in when both cerebral abscess and meningitis are in the differential diagnosis. Early recognition and prompt intervention is crucial to prevent severe outcomes, such as elevations in intracranial pressure, brain herniation, sepsis, reinfection, and death. It is important to consider atypical pathogens, such as *F necrophorum*, to identify pathogens quickly and accurately, allowing for optimization of antimicrobial management. Our case illustrates the importance of comprehensive diagnostic and management approaches by a multidisciplinary care team for patients faced with complex and illusive pathophysiology.
